# Targeted Biopsy Based on ADC Map in the Detection and Localization of Prostate Cancer: A Feasibility Study

**DOI:** 10.1002/jmri.23908

**Published:** 2013-05

**Authors:** Yuji Watanabe, Masako Nagayama, Tohru Araki, Akito Terai, Akira Okumura, Yoshiki Amoh, Takayoshi Ishimori, Satoru Nakashita, Yoshihiro Dodo

**Affiliations:** 1 Department of Radiology, Kurashiki Central HospitalKurashiki, Japan; 2 Araki Urologic ClinicKurashiki, Japan; 3 Department of Urology, Kurashiki Central HospitalKurashiki, Japan

**Keywords:** magnetic resonance imaging, prostate cancer, targeted biopsy, transition zone, diffusion-weighted imaging, apparent diffusion coefficient

## Abstract

**Purpose:**

To investigate the feasibility of targeted biopsy based on an apparent diffusion coefficient (ADC) map in the detection and localization of prostate cancer.

**Materials and Methods:**

This study included 288 consecutive patients with high or increasing serum prostate-specific antigen (PSA) levels who underwent prostatic magnetic resonance imaging (MRI) examination with an ADC map. Four core-targeted biopsies of low ADC lesions were performed under transrectal-ultrasound guidance with reference to ADC map. The positive predictive values (PPVs) of low ADC lesions were calculated and compared for the peripheral zone (PZ), transition zone (TZ), and anterior portion, respectively. Comparisons of ADC values and sizes between malignant and nonmalignant lesions were also performed.

**Results:**

A total of 313 low ADC lesions were detected in 195 patients and sampled by targeted biopsies. The PPVs were 55.3% (95% confidence interval [CI]: 50–61) in total, 61.0% (95% CI: 53–69) for PZ, 50.6% (95% CI: 43–58) for TZ, and 90.9% (95% CI: 81–100) for the anterior portion. The most common nonmalignant pathology of low ADC lesions was hyperplasia, followed by chronic prostatitis. There were significant differences in ADC values and sizes between malignant and nonmalignant low ADC lesions.

**Conclusion:**

Targeted biopsies could be capable of detecting cancers well wherever they may be in the prostate, although the PPVs varied depending on the location of low ADC lesions.

MAGNETIC RESONANCE IMAGING (MRI) of the prostate gland with endorectal coil or pelvic phased array coil has been widely used to detect and localize malignant lesions that mainly occur in the peripheral zone (1-3). Recently, a number of investigators have reported the potential usefulness of diffusion-weighted imaging (DWI) and an apparent diffusion coefficient (ADC) map for detecting prostate cancer, which shows lower ADC than a normal peripheral zone and a nonmalignant transition zone (2, 4–8).

Transrectal ultrasound (TRUS)-guided prostate biopsies have been used to detect prostate cancer. Systematic prostate biopsies to take six cores were first recommended, and much of the recent work to improve cancer detection has focused on increasing the total number of biopsies with the inclusion of additional laterally placed biopsies of the peripheral zone (9–13). It was reported that prostate biopsies, when additional targeted biopsy cores were given to sample low ADC lesions, had possibilities to yield a high cancer detection rate and to detect cancer which might be missed only with systematic biopsies focusing on the peripheral zone (8). In order to target low ADC lesions precisely, real-time TRUS combined with previously acquired MRI was reported to be feasible and useful to guide prostate biopsies (14). This technique, however, requires special equipment and fusion software, which might limit its availability. Even simple TRUS-guided prostate biopsies, when targeted biopsy cores are focused on highly suspicious areas according to MRI information of low ADC lesions, could successfully take samples from the targeted lesions to detect and localize prostate cancer. This could also allow for evaluating low ADC lesions in the context of tumor location and size.

In this feasibility study we aimed to explore the effectiveness of targeted prostate biopsies based on ADC map findings in the detection and localization of prostate cancer and to investigate what low ADC lesions represent in histopathology.

## MATERIALS AND METHODS

### Patient Population

This prospective study enrolled 288 consecutive patients (mean age: 70.9 years old, range 50–88) suspected of having prostate cancer because of either high or increasing serum PSA levels (mean level: 9.7 ng/mL, range 2.8–49.2 ng/mL) between August 2004 and March 2007. Before being enrolled in this study each patient gave written informed consent to be involved in the investigation, which was approved by the Institutional Review Board. All the patients underwent MR examination with a DWI and ADC map.

### MRI

MRI was performed on a 1.5 T superconductive magnet system (Gyroscan Achieva; Philips Medical Systems, Best, Netherlands) with the synergy cardiac coil. After initial T1-weighted localizing images were obtained, MR images of the entire prostate gland and the seminal vesicle in the transaxial direction were acquired with DWI and 3D T2-weighted turbo spin echo (TSE) imaging.

DWI was performed with a spin-echo echo-planar-imaging sequence in the straight transaxial direction. The scan parameters were b-values of 0 and 600 s/mm^2^, TR/TE 6300/50, section thickness of 2.5 mm without intersection gap, field of view of 350 mm, matrix of 144 × 256 that was interpolated to 256 × 256, voxel size of 1.37 × 1.37 × 2.5 mm^3^, seven signal acquisition, and scan time of 5 minutes 21 seconds. The ADC maps with 2.5-mm-thick sections without intersection overlap were constructed from ADC values calculated from signal intensity data obtained in the DWIs with b-values 0 and 600 s/mm^2^. Then, using the 2.5-mm-thick ADC maps, the transaxial, coronal, and sagittal ADC maps were reconstructed with 4-mm-thick gapless sections to match 4-mm-thick sections of the following T2-weighted images.

For T2-weighted imaging, thin-section high-spatial-resolution 3D T2-weighted TSE images were obtained in the straight transaxial plane with the following parameters: TR/TE 1500/150 msec; echo train length of 61, section thickness of 1.4 mm with intersection overlap of 0.7 mm; field of view of 200 mm; matrix of 224 × 512 that was interpolated to 512 × 512; reconstructed voxel size of 0.4 × 0.4 × 0.7 mm^3^; and number of excitations of two. Total acquisition time was 7 minutes 27 seconds. Then transaxial, coronal, and sagittal images were reconstructed with 4-mm-thick gapless sections, which provided anatomical details of the prostate gland.

### Image Analysis

All images were analyzed by the two radiologists who were aware of the patients' serum PSA levels, with use of soft-copy reading on an electronic workstation (ShadeQuest; Yokogawa Medical Solutions, Tokyo, Japan). To detect low ADC lesions, the readers first evaluated ADC maps on the display in the standard grayscale mode with the window width and level set at 2400 and 1350, respectively. Then areas of ADC values of 1.35 × 10^−3^ mm^2^ /sec appeared as a median gray color, which facilitated visually detecting low ADC lesions of 5 mm or greater with ADC values of 1.35 × 10^−3^ mm^2^ /sec or less. ADC values of the low ADC lesions were measured with elliptical or multiangular user-defined regions-of-interest (ROIs) drawn over the low ADC lesions. The diameter of each ROI was about 70%–80% of the diameter of the lesion. Then the short and long diameters of the lesions were measured on the corresponding T2-weighted images which were matched with the ADC maps using a slice-location function. The low ADC lesions were also visually evaluated on T2-weighted images in terms of the shape, the signal intensity homogeneity, and the capsule.

The criteria to select low ADC lesions for targeted biopsy were the following ADC map features: nodular and homogeneous appearance of round, oval, or crescent shape, ADC value of 1.35 × 10^−3^ mm^2^ /sec or less, short diameter of 5 mm or greater. Then exclusion criteria to eliminate benign lesions were applied for the selected lesions, using the characteristics on T2-weighted images indicative of hyperplastic nodules: inhomogeneous signal intensity with high-intensity spots, round shape and smooth margin with hypointense pseudocapsule (15, 16). Special caution was taken not to mistake normal central zone for low ADC lesion to be targeted at biopsy (17), because the normal central zone mimics malignant lesion by its low ADC value. T2-weighted images in both coronal and transaxial planes were used for identifying the normal central zone with the following typical findings: symmetric anatomical location overlying transition zone and crescent shape surrounding ejaculatory duct with homogeneous T2-low signal intensity.

Then the low ADC lesions selected finally for the following targeted biopsy were recorded with regard to the location, shape, and size (short and long diameters). To report the location of the low ADC lesions, the transition and peripheral zones were segmented into the upper, middle, and lower thirds. The left and right sides were separated by the median sagittal plane through the verumontanum. The direction in the transverse plane was also described as a clock time. Special attention was paid to the “anterior portion” indicating the portion anterior to the prostatic urethra including the transition and peripheral zones in the direction from 10 to 2 o'clock, in which cancer foci would be missed only with conventional systematic biopsies.

### Biopsy Protocol

Biopsies were taken with a spring-loaded Pro-Mag Ultra automatic biopsy instrument (MD TEC, Denmark) and an 18 G needle under guidance of transrectal ultrasonography (TRUS). The patients who were found to have low ADC lesions underwent a 12-core biopsy to take eight systematic biopsy cores and four targeted biopsy cores. Systematic biopsies were performed with six lateral cores sampling from the lateral peripheral zones at the upper, middle, and lower segments, and two paramedian cores sampling from the mid-parasagittal peripheral and transition zones at the middle segment.

Then four additional targeted biopsy cores were focused on the low ADC lesions and directed to the suspicious foci according to the MR reports. Targeted biopsies were performed under TRUS guidance as follows: when a low ADC lesion to be targeted was detected as a low-echoic lesion on TRUS, biopsy cores for the lesion were readily obtained under TRUS guidance. When a targeted low ADC lesion was not detected on TRUS, targeted biopsies were taken from the segment in the direction of the clock time according to the MR information of the target low ADC lesion. A maximum of four largest low ADC lesions were sampled in each patient. When a patient had only one low ADC lesion to be targeted, all four biopsy cores were directed toward it. When a patient had two low ADC lesions, two biopsy-cores were directed toward each targeted lesion. When a patient had three low ADC lesions, two biopsy cores were directed toward the largest targeted lesion, and one biopsy core toward each of the others. When a patient had four low ADC lesions a single biopsy-core was directed toward each lesion.

Targeted biopsy cores of low ADC lesions were usually taken from the posterior–anterior straightforward directions and/or outside–inside oblique directions. Especially for an anterior portion lesion, targeted biopsy cores were taken from both right and left outside–inside oblique directions to avoid taking sample from the urethra.

In each patient, all the biopsy specimens obtained were numbered and the locations and biopsy courses were recorded on the scheme of the prostate gland. Each numbered biopsy specimen was marked at the rectal-side tip with black ink and examined histologically.

### Histological Evaluation of Biopsy Specimens

For each patient, biopsy specimens served as the reference standard. Histological results were reported by the experienced pathologists as to the presence or absence of prostate cancer and other nonmalignant conditions such as hyperplasia with or without atrophic degeneration, atypical adenomatous or basal-cell hyperplasia, prostatic intraepithelial neoplasia (PIN), chronic prostatitis, granulomatous prostatitis, etc.

When prostate cancer was histologically diagnosed, the following details of the biopsy core were also reported: the sample number of positive result, the location of the cancer on the core, distance from the rectal-side tip, the proportion in length of the cancer occupying in the positive sample core, and the differentiation type of cancer. A Gleason score (GS) of the cancer was also reported.

### Statistical Analysis

Histopathology was the standard of reference, and all histopathological results were included in the case record form. Among the lesion locations of peripheral zone, transition zone, and anterior portion, positive predictive values (PPVs) of the low ADC lesions were calculated and compared. Among the low ADC lesions with positive biopsy results the relationship between the ADC values and the GS was also examined. Statistical analysis was performed with a Kruskal–Wallis test. Comparisons of ADC values and sizes between malignant and nonmalignant lesions were performed with Mann–Whitney's *U*-test. In every statistical analysis, significance was considered to exist when the *P*-value was less than 0.05.

## RESULTS

Among the 288 patients studied, 195 patients (mean age: 71.4 years old, range 50–88; mean PSA level 9.9 ng/mL, range 2.9–49.2) were shown to have low ADC lesions and underwent prostate biopsies. In the 195 patients, 313 low ADC lesions were examined with targeted biopsies; 38 lesions (12%) were seen on TRUS and 275 lesions (88%) were biopsied in an ADC map-guided fashion without visualization on TRUS. As shown in Table 1, the mean ADC value of the low ADC lesions was 1.04 × 10^−3^ mm^2^ /sec and the mean long diameter and the standard deviation was 13.4 ± 7.0 mm with range 5–51 mm. The most common location that contained a low ADC lesion was the transition zone (*n* = 172, 55.0%, 95% confidence interval [CI]: 49–60) followed by the peripheral zone (*n* = 141, 45.0%, 95% CI: 40–51). The anterior portion included 33 lesions (10.5%, 95% CI: 7–14). The lesion size was significantly larger and the ADC value was also significantly lower for the anterior portion than the peripheral and the transition zones (*P* < 0.005).

**Table 1:** Characteristics of Low-ADC Lesions Sampled by Targeted Biopsies

Location	Number of low ADC lesions	% (95%CI)	Size (cm) mean ± SD	ADC (×10^−3^ mm^2^ /sec) mean ± SD
Peripheral zone	141	45.0 (40–51)	12.6 ± 6.6*	1.08 ± 0.18*
Transition zone	172	55.0 (49–60)	13.7 ± 7.1*	1.01 ± 0.16*
Anterior Portion	33	10.5 (7–14)	17.1 ± 8.0*	0.93 ± 0.19*
Overall	313	100	13.4 ± 7.0	1.04 ± 0.17

* *P* < 0.005 with Kruskal–Wallis test.

**Figure 1:**
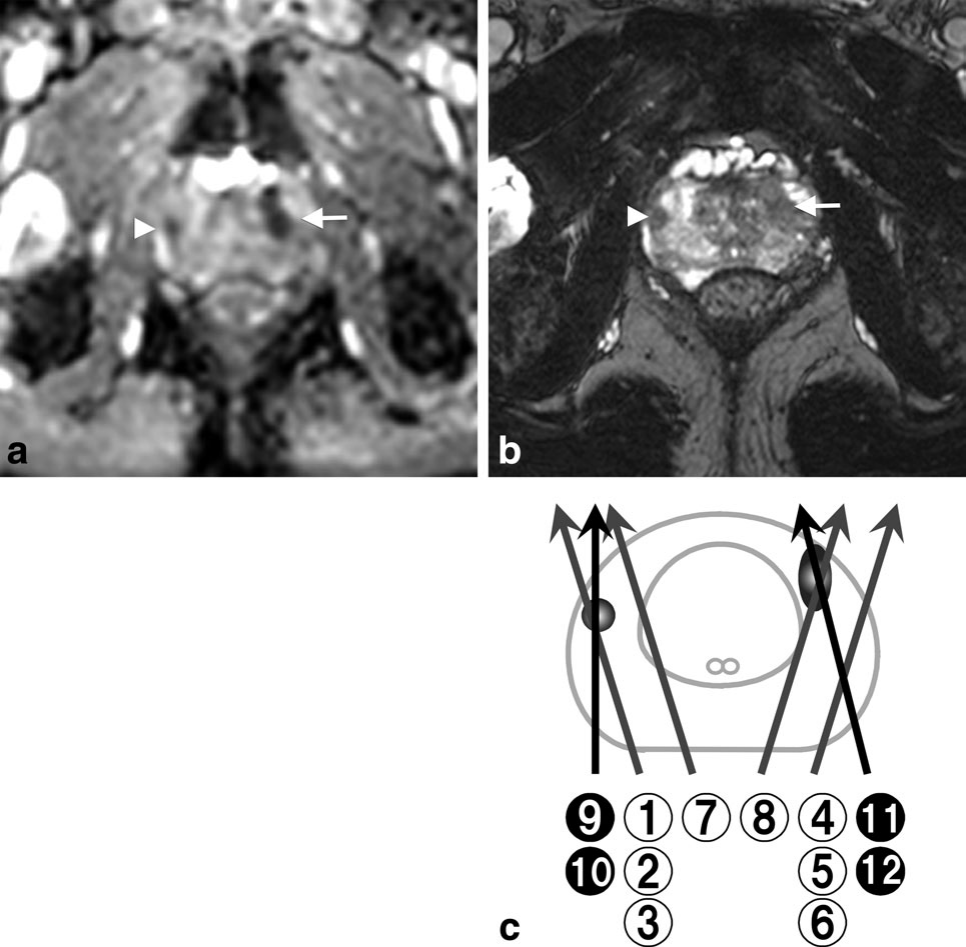
Prostate cancer in the left and right middle peripheral zones in a 73-year-old patient with high serum PSA level of 7.0 ng/mL. **a:** Apparent diffusion coefficient (ADC) map. **b:** T2-weighted image. **c:** Scheme of systematic and targeted biopsies. ADC map (a) demonstrates an oval low ADC (0.80 × 10^−3^ mm^2^ /sec) lesion (arrow) in the left middle peripheral zone and a small round low ADC (1.12 × 10^−3^ mm^2^ /sec) lesion (arrowhead) in the right middle peripheral zone. Both the left peripheral zone lesion (arrow) and the small right peripheral zone lesion (arrowhead) show low signal intensity on T2-weighted image (b). The scheme of prostate biopsy (c) demonstrates the number, location, and course of all the biopsy specimens including eight systematic biopsy cores (gray arrows) and four targeted biopsy cores (black arrows). Histological examination proves both the peripheral zone lesions to be moderately differentiated adenocarcinoma in the targeted biopsy specimens.

**Figure 2:**
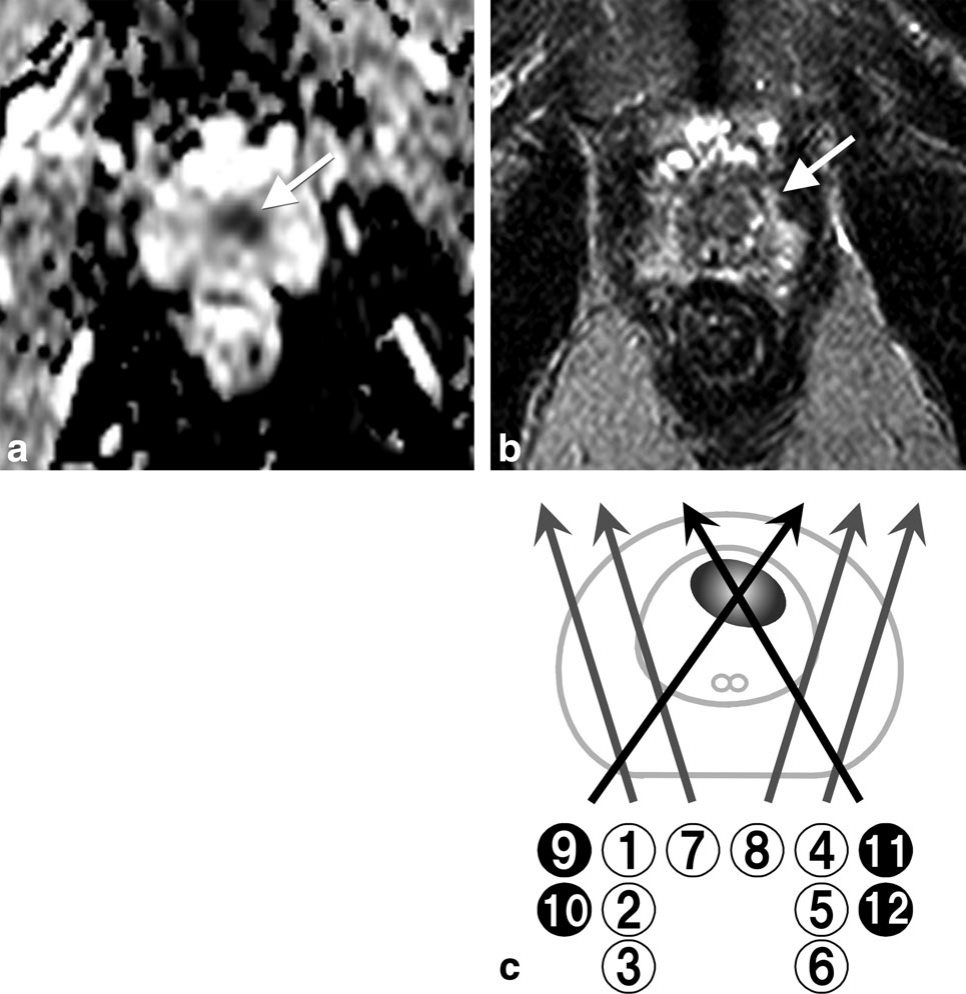
Prostate cancer in the apical transition zone in a 72-year-old patient with high serum PSA level of 4.01 ng/mL. **a:** Apparent diffusion coefficient (ADC) map. **b:** T2-weighted image. **c:** Scheme of systematic and targeted biopsies. ADC map (a) demonstrates an oval low ADC (0.62 × 10^−3^ mm^2^ /sec) lesion (arrow) in the anterior apical transition zone. The lesion (arrow) shows intermediate signal intensity on T2-weighted image (b). The scheme of prostate biopsy (c) demonstrates the number, location, and course of all the biopsy specimens including eight systematic biopsy cores (gray arrows) and four targeted biopsy cores (black arrows). Histological examination proves the lesion to be well-differentiated adenocarcinoma only in the targeted biopsy specimens.

**Figure 3:**
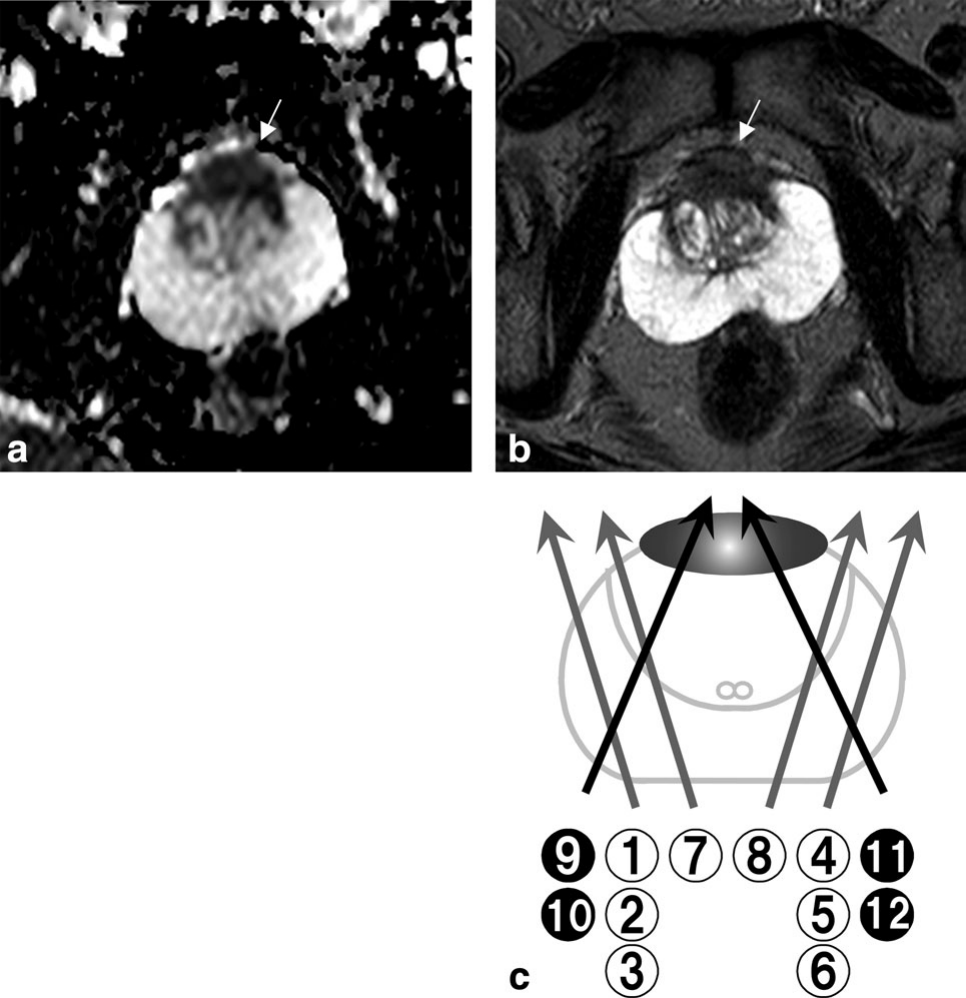
Prostate cancer in the anterior middle transition zone and fibromuscular stroma in a 73-year-old patient with high serum prostate-specific antigen (PSA) level of 18.3 ng/mL. **a:** Apparent diffusion coefficient (ADC) map. **b:** T2-weighted image. **c:** Scheme of systematic and targeted biopsies. ADC map (a) demonstrates a crescent-shaped low ADC (0.72 × 10^−3^ mm^2^ /sec) lesion (arrow) in the anterior middle transition zone and fibromuscular stroma of the prostate gland. The lesion (arrow) shows low signal intensity on T2-weighted image (b). The scheme of prostate biopsy (c) demonstrates the number, location, and course of all the biopsy specimens including eight systematic biopsy cores (gray arrows) and four targeted biopsy cores (black arrows). Histological examination reveals moderately-differentiated adenocarcinoma only in the targeted biopsy specimens.

Of all the 313 low ADC lesions examined by targeted biopsies, there were 173 cancer foci (55.3%, 95% CI: 50–61) detected in 121 patients (Table 2). The location of the 173 cancer lesions was the peripheral zone in 86 (49.7%) and the transition zone in 87 (50.3%) (Figs. 1, 2). As a unique site of cancer, 30 cancer foci (17.3%) were detected in the “anterior portion” (Fig. 3). The PPVs of the targeted biopsies sampling the low ADC lesions were 55.3% (95% CI: 50–61) in total, 61.0% (95% CI: 53–69) for the peripheral zone, 50.6% (95% CI: 43–58) for the transition zone, and 90.9% (95% CI: 81–100) for the anterior portion (Table 2). The PPVs were significantly different according to the location of the lesion (*P* < 0.05, Kruskal–Wallis test). The systematic biopsy cores taken from segments that did not include any low ADC lesions unexpectedly found 12 cancer foci in eight patients (4%).

**2:** Positive Predictive Values of Low-ADC Lesions Examined by Targeted Biopsies

	Positive predictive value		
	
Location	Number	%	95% CI
Peripheral zone	86/141	61.0*	53–69
Transition zone	87/172	50.6*	43–58
Anterior portion	30/33	90.9*	81–100
Overall	173/313	55.3	50–61

* *P* < 0.005 with Kruskal-Wallis test.

The GS was determined in 165 out of the 173 cancer foci, and could not be determined in eight cancer foci because they were too small. The mean ADC values and standard deviations of each GS category were 0.98 ± 0.19 for GS 5, 1.03 ± 0.17 for GS 6, 0.99 ± 0.17 for GS 7, 0.94 ± 0.15 for GS 8, and 0.90 ± 0.14 (×10^−3^ mm^2^ /s) for GS 9 (Table 3). Although there seemed to be a tendency that ADC values of cancer foci with GS 8 or 9 might be lower than those with GS 7 or smaller, no significant differences were observed.

**3:** ADC Values of Cancer Foci According to the Gleason Scores

	Gleason score					
		
	5	6	7	8	9	*P*-value
ADC values (mean ± SD)	0.98 ± 0.19	1.03 ± 0.17	0.99 ± 0.17	0.94 ± 0.15	0.89 ± 0.13	0.097
Number of cancer foci	38	16	64	28	19	

*P*-value with Kruskal-Wallis test.

### Nonmalignant Lesions

**Figure 4:**
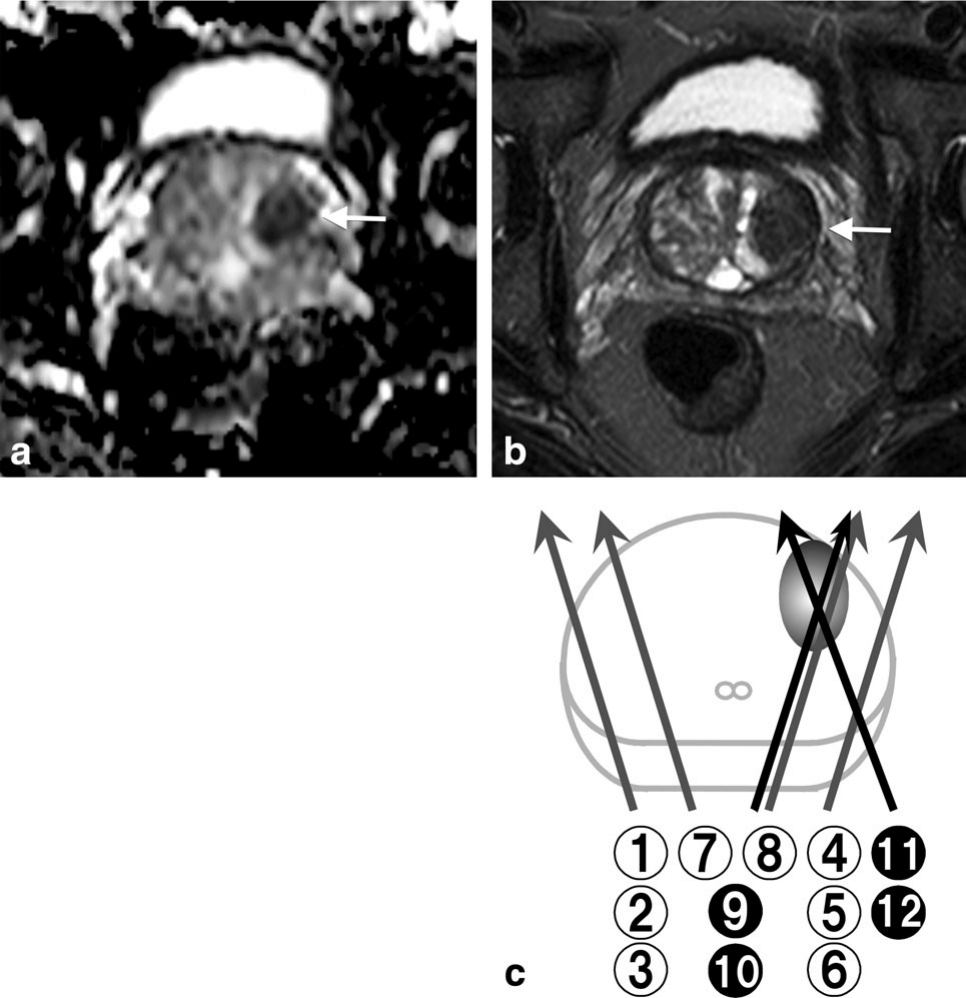
Benign hyperplastic nodule in the transition zone in a 69-year-old patient with high serum PSA level of 9.2 ng/mL. **a:** Apparent diffusion coefficient (ADC) map. **b:** T2-weighted image. **c:** Scheme of systematic and targeted biopsies. ADC map (a) demonstrates a large oval low ADC (1.09 × 10^−3^ mm^2^ /sec) lesion (arrow) in the basal transition zone. The lesion (arrow) shows low signal intensity on T2-weighted image (b). The scheme of prostate biopsy (c) demonstrates the number, location, and course of all the biopsy specimens including eight systematic biopsy cores (gray arrows) and four targeted biopsy cores (black arrows). Histological examination proves the lesion to be hyperplasia in the targeted biopsy specimens.

**Figure 5:**
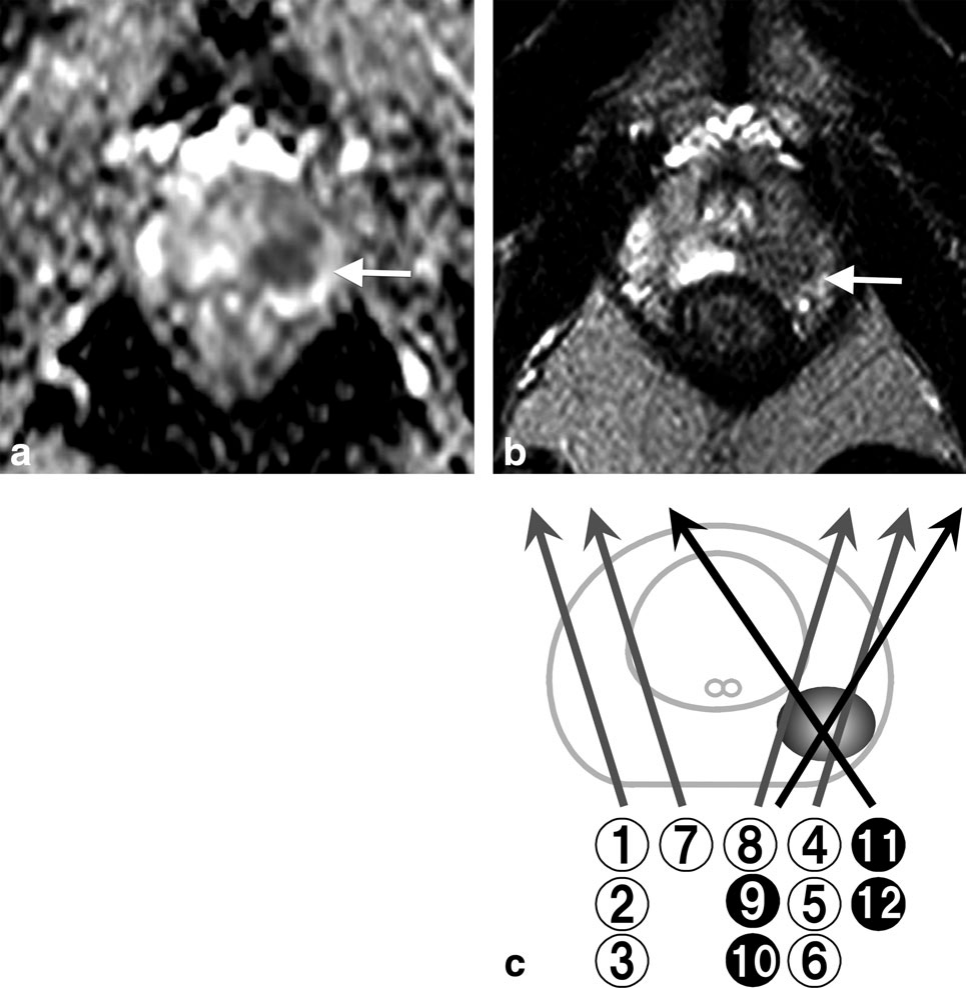
Granulomatous prostatitis in the peripheral zone in a 60-year-old patient with high serum PSA level of 4.6 ng/mL. **a:** Apparent diffusion coefficient (ADC) map. **b:** T2-weighted image. **c:** Scheme of systematic and targeted biopsies. ADC map (a) demonstrates a large oval low ADC (1.07 × 10^−3^ mm^2^ /sec) lesion (arrow) in the apical transition zone. The lesion (arrow) shows low signal intensity on T2-weighted image (b). The scheme of prostate biopsy (c) demonstrates the number, location, and course of all the biopsy specimens including eight systematic biopsy cores (gray arrows) and four targeted biopsy cores (black arrows). Histological examination proves the lesion to be granulomatous prostatitis in the targeted biopsy specimens.

Of all the 313 low ADC lesions, 140 lesions (44.7%) were found to be nonmalignant, including 55 lesions (39.3%) in the peripheral zone and 85 lesions (60.7%) in the transition zone (Table 4). Histopathologically, hyperplasia with or without atrophic degeneration was the most common (66%, 95% CI: 59–74), followed by atypical basal cell hyperplasia (9%, 95% CI: 5–14), chronic prostatitis (8%, 95% CI: 4–13), high-grade prostatic intraepithelial neoplasia (PIN) (7%, 95% CI: 3–11), granulomatous prostatitis (3%, 95% CI: 0–6), atypical adenomatous hyperplasia (2%, 95% CI: 0–5), and unspecified benign lesions (4%, 95% CI: 0–7) (Figs. 4, 5).

**4:** Histopathological Findings of Nonmalignant Low-ADC Lesions Sampled by Targeted Biopsies

	Location								
	
	Overall			Peripheral zone			Transition zone		
			
Histopathological findings	Number of lesions	%	95% CI	Number of lesions	%	95% CI	Number of Lesions	%	95% CI
Hyperplasia with or without atrophic degeneration	93	66	59–74	27	49	36–62	66	77	69–87
ABH	13	9	5–14	5	9	1–17	8	9	3–16
High-grade PIN	10	7	3–11	6	11	3–19	4	5	0–9
Chronic prostatitis	12	8	4–13	10	18	8–28	2	2	0–6
Granulomatous prostatitis	4	3	0–6	4	7	0–14	0	0	0–0
AAH	3	2	0–5	2	4	0–9	1	1	0–3
Unspecified benign lesion	5	4	0–7	1	2	0–5	4	5	0–9
Total	140	100		55	100		85	100	

ABH: atypical basal-cell hyperplasia; PIN: prostatic intraepithelial neoplasia; AAH: atypical adenomatous hyperplasia.

### Comparison of ADC Value and Lesion Size Between Malignant and Nonmalignant Lesions

There were significant differences in ADC values and lesion sizes between malignant and nonmalignant low ADC lesions (Table 5). Malignant lesions showed lower ADC value and larger size than nonmalignant lesions (*P* < 0.001).

**5:** Comparisons of ADC Value and Lesion Size Between Malignant and Non-malignant Low-ADC Lesions Examined by Targeted Prostate Biopsies

	Malignant Lesion (n = 173)	Non-Malignant Lesion (n = 140)	p-value
Mean ADC (SD)	0.97 (0.17)	1.12 (0.14)	p < 0.001
Mean Size (SD) (mm)	15.0 (7.6)	11.5 (5.7)	p < 0.001

ADC: Apparent Diffusion Coefficient p-value with Mann-Whitney's U-test

## DISCUSSION

The prostate cancer detection rate has been reported to vary depending on biopsy protocols and number of biopsy cores. Standard peripheral zone biopsies to take six cores historically yielded a cancer detection rate of 30%–35% (18). Initial saturation biopsy with 21–24 biopsy cores and conventional extended biopsy to take 10–12 cores yielded higher detection rates up to 44.6% and 51.7%, respectively (9, 19). Targeted biopsy protocol taking 10–12 cores, which had 2–4 targeted biopsy cores directed to low ADC lesions in addition to 8-core systematic biopsy was reported to show a 70.1% cancer detection rate (8). This feasibility study has demonstrated satisfactory lesion-based PPV of 55.3%, and suggested that even simple TRUS-guided prostate biopsies, when focused on highly suspicious areas according to MRI information of low ADC lesions, could successfully take samples from the targeted lesions. Furthermore, this lesion-by-lesion study may suggest that targeted biopsies could detect cancers well wherever they may be in the prostate gland, although the PPVs varied depending on the location of low ADC lesions.

The anterior portion seems to be very special and unique in the detection of cancer because those cancers require additional sets of biopsies before detection (20). Takashima et al (21) found that more than half of the apical tumors were located in the anterior half of the prostate, an area of the prostate referred to as the “anterior peripheral zone.” Bott et al (20) reported that 21% of prostate cancers were located in the anterior zone and that those cancers required additional sets of biopsies before detection. Wright and Ellis (22) showed the importance of anterior apical biopsy, because the most common site of a single tumor was the anterior apex and several cancers would have been missed by standard peripheral zone biopsies. Therefore, it is important to biopsy the anterior portion of the prostate for diagnostic purposes. This feasibility study demonstrated that most of the low ADC lesions in the anterior portion proved to be malignant, with a high PPV of 90.9%. These results may suggest that the anterior prostate biopsy could be very effectively incorporated when ADC maps reveal low ADC lesions in the anterior portion.

The transition zone has not been focused on by standard biopsies because of a low prevalence of cancer, which may be attributed to the recognition that prostate cancers are frequently missed on initial biopsies (23). Detection of transition zone cancer with the ADC map has been highlighted by some reports (17, 24). It has been shown that the transition zone harbors cancer in up to 25% of radical prostatic specimens (25–27). Detection of prostate cancer in the transition zone has been much more difficult because the signal intensity of transition zone cancer can have a heterogeneous appearance similar to that of hyperplasia on T2-weighted images (26, 28). The capability of detecting a cancer in the transition zone is a great advantage of ADC maps over conventional T2-weighted images. Our study demonstrated that the targeted biopsies of low ADC lesions detected as many cancer foci in the transition zone as in the peripheral zone. The PPV for transition zone cancer was as high as 50.6%, but lower than that for peripheral zone cancer. The false-positive results of low ADC lesions in the transition zone were exclusively found in hyperplastic nodules. Differentiation between benign and malignant lesions seems to be limited on the basis of ADC value alone. Although there was a tendency observed in this study that cancer lesions showed lower ADC values than nonmalignant lesions, including hyperplastic nodules, considerable overlap was observed in ADC values between malignant and nonmalignant lesions. In order to increase the PPV by reducing the number of false-positives, it should be necessary to apply more anatomical details on T2-weighted images for the selection criteria. As shown in the previous reports, findings supporting the diagnosis of a transition zone cancer include 1) a homogeneous low-signal-intensity; 2) poorly defined or spiculated lesion margins; 3) lack of a low-signal-intensity rim; 4) interruption of the transition zone-to-peripheral zone boundary of low signal intensity); 5) urethral or anterior fibromuscular stromal invasion; and 6) lenticular shape (15, 29). In addition, a combination with T2-weighted imaging and dynamic contrast-enhanced imaging could be necessary to yield higher sensitivities and specificities in the detection of transition zone cancer by incorporating information from different aspects such as morphology, T2 value, and vascular properties (5, 6, 15–17, 29, 30).

For the peripheral zone, targeted biopsy results yielded a high PPV of 61%. Yoshimitsu et al (17) reported that interpretation of T2-weighted images along with ADC maps improved sensitivity up to 71% but did not increase specificity in their retrospective study. The false-positives included hyperplasia with atrophic degeneration, chronic prostatitis, granulomatous prostatitis, high-grade PIN etc. (31), which mimicked cancer on both ADC maps and T2-weighted images. To solve this issue, several remedies to offer better detection of peripheral zone cancer, such as the logistic regression analysis (32), wash-in rate on the basis of dynamic contrast-enhanced imaging (33), and MR spectroscopy (34), have been proposed and should be further investigated.

Another major problem in the detection of peripheral zone cancer has been reported to be sparsely spread cancer (35). In this feasibility study, a small number (4%) of patients had cancer found unexpectedly in segments not to include low ADC lesions. Most such false-negative cancer lesions were reported to be sparsely spread cancer that limits MRI detection (35), and reported to be so-called “insignificant cancers” characterized histologically as being low volume (<0.5 cm^3^ ) and low grade (GS < 7) and might not need radical or potentially harmful treatment (8, 9, 13, 36).

The ADC values were reported to correlate with GS (37). Although there seemed to be a trend of decreasing ADC values with increasing GS in our study, no significant difference was observed between ADC values and GS. ADC values obtained using a b-value 0 and 600 DWI could be theoretically higher than that obtained using b-value 100 and 600 DWI, because b-value 0 images include both perfusion and diffusion. This may contribute to our results showing no significant difference between ADC value and GS. From a viewpoint of clinical practice, however, it would still have some advantages to use b-value 0 over b-value 100 as a baseline DWI for the measurement of ADC value such as shorter examination time and reduction of motion artifacts.

The small size of cancer foci might be another reason why cancers are frequently missed on initial biopsy. In this study, even a 5-mm-sized lesion, especially in the peripheral zone, can be clearly demonstrated on an ADC map. A high-quality ADC map should be crucial to detect and localize small-sized cancer foci. In order to obtain high-quality ADC maps derived from DWI without the use of an endorectal coil, several technical considerations in DWI should include a thin slice, high spatial resolution, short TE, high number of signal acquisitions, and relatively small b-value (b = 600 s/mm^2^ ) used (8). No special instructions were given to the patients prior to MRI regarding diet and bowel preparation. By taking several biopsy cores focused on the lesion itself or segment in the direction as a clock time of a low ADC lesion based on the ADC map information, even 5-mm-sized cancer foci could be detected and localized accurately. Although these cancers might not need radical or potentially harmful treatment (38), identifying them at the time of biopsy remains a critical issue.

This study has an inherent limitation of possible sampling error from the low ADC lesions. According to the MR reports, targeted biopsies were carefully designed and performed to obtain samples from the lesions or segments of the suspicious foci. Most (88%) of the low ADC lesions were not seen on TRUS and were biopsied in an ADC map-guided fashion without visualization on TRUS. Thus, the risk of sampling error cannot be ignored. The ultrasound-guided biopsy performed under real-time ultrasonography-MRI fusion-guidance (14) and MR-guided biopsy could overcome this problem, but requires special equipment and/or fusion software, which might limit its availability. In reality, more targeted biopsy cores focused on a low ADC lesion could be readily performed to increase the probability for precise sampling of the target lesion. Furthermore, it should be investigated whether only targeted biopsies without backup of systematic biopsy could offer better detection of clinically significant cancer in patients with suspicious low ADC lesion.

In summary, this study has demonstrated the feasibility and usefulness of targeted biopsies together with ADC maps in the detection and localization of prostate cancer. An ADC map in combination with targeted biopsies could improve detection and localization of prostate cancer especially in the anterior portion, although low ADC lesions include not only malignant but also benign or borderline lesions.
